# Outcomes in older adults with metastatic esophageal and gastric carcinoma treated with palliative chemotherapy

**DOI:** 10.1093/oncolo/oyae190

**Published:** 2024-07-24

**Authors:** Xin Wang, Michael J Allen, Osvaldo Espin-Garcia, Chihiro Suzuiki, Yvonne Bach, Elan Panov, Lucy X Ma, Raymond W Jang, Eric X Chen, Gail E Darling, Jonathan Yeung, Carol J Swallow, Savtaj Singh Brar, Sangeetha Kalimuthu, Rebecca Wong, Patrick Veit-Haibach, Elena Elimova

**Affiliations:** Division of Medical Oncology, Princess Margaret Cancer Centre, University Health Network, Toronto ON, Canada; Division of Medical Oncology, Princess Margaret Cancer Centre, University Health Network, Toronto ON, Canada; Department of Biostatistics, Princess Margaret Cancer Centre, University Health Network, Toronto, ON, Canada; Division of Medical Oncology, Princess Margaret Cancer Centre, University Health Network, Toronto ON, Canada; Division of Medical Oncology, Princess Margaret Cancer Centre, University Health Network, Toronto ON, Canada; Division of Medical Oncology, Princess Margaret Cancer Centre, University Health Network, Toronto ON, Canada; Division of Medical Oncology, Princess Margaret Cancer Centre, University Health Network, Toronto ON, Canada; Division of Medical Oncology, Princess Margaret Cancer Centre, University Health Network, Toronto ON, Canada; Division of Medical Oncology, Princess Margaret Cancer Centre, University Health Network, Toronto ON, Canada; Division of Thoracic Surgery, Toronto General Hospital, University Health Network, Toronto, ON, Canada; Division of Thoracic Surgery, Toronto General Hospital, University Health Network, Toronto, ON, Canada; Department of Surgical Oncology, Princess Margaret Cancer Centre, University Health Network, Toronto, ON, Canada; Department of Surgery, Mount Sinai Hospital, Toronto, ON, Canada; Department of Surgery, Mount Sinai Hospital, Toronto, ON, Canada; Division of Pathology, Toronto General Hospital, University Health Network, Toronto, ON, Canada; Division of Radiation Oncology, Princess Margaret Hospital, University Health Network, Toronto, ON, Canada; Joint Department of Medical Imaging, Toronto General Hospital, University Health Network, Toronto, ON, Canada; Division of Medical Oncology, Princess Margaret Cancer Centre, University Health Network, Toronto ON, Canada

**Keywords:** gastric cancer, esophageal cancer, geriatric oncology, palliative chemotherapy

## Abstract

**Background:**

The incidence of esophageal and gastric carcinoma (GEC) in elderly patients is increasing, yet patients ≥75 years have historically been underrepresented in clinical trials. We sought to investigate palliative chemotherapy administration patterns and survival outcomes in older adults.

**Materials and Methods:**

A retrospective analysis identified patients aged 65-74 (young-old) and ≥75 years (older-old) diagnosed with advanced GEC. Patient and tumor characteristics were recorded, with descriptive analysis, time-to-event data analysis using Kaplan-Meier curves and multivariate Cox proportional hazards regression analysis performed.

**Results:**

One hundred and ninety-eight “young-old” and 109 ‘older-old’ patients were identified. Patient characteristics were similar between groups except for Charlson Co-morbidity Index (CCI), with lower co-morbidities in the “young-old” compared to “older-old” cohort (*P* < .001; CCI = 0 in 103 (52%) “young-old” vs 31 (28%) “older-old”). The primary diagnosis in both groups was adenocarcinoma. 119 (60%) “young-old” and 25 (23%) “older-old” patients received chemotherapy (*P* < .001). Performance status was the primary explanation for chemotherapy non-receipt in both cohorts; age was the explanation in 21 (25%) “older-old” patients and none in the “young-old” patients. PFS for first-line systemic therapy in “young-old” patients was 6.4 (95% CI 5.9-7.6) versus 7.5 months (95% CI 5.1-11.3) in “older-old” patients (*P* = .69) whilst respective OS was 12.3 (95% CI 10.1-15.5) and 10.4 months (95% CI 9.0-14.6) (*P* = .0816). Toxicity prompted chemotherapy cessation in 17 (15%) “young-old” and 3 (13%) “older-old” patients (*P* = .97). Multivariate analysis identified CCI and ECOG performance status as predictive for PFS and OS, respectively. No causative relationship was identified with other variables.

**Conclusion:**

Our study of real-world older-adults show that significant number of “older-old” patients with GEC do not receive chemotherapy. Among “older-old” adults who do receive systemic therapy, outcomes are comparable; this underscores the importance of geriatric assessment-guided care and suggests that age alone should not be a barrier to receipt of chemotherapy in patients with advanced GEC.

## Teaser

The incidence of esophageal and gastric carcinoma in elderly patients is increasing, yet patients ≥75 years have historically been underrepresented in clinical trials. This study investigated palliative chemotherapy administration patterns and survival outcomes in older adults.

**Implications for practice**

Gastroesophageal malignancies are a leading cause of cancer-related deaths and morbidity and are increasingly being diagnosed in older adults. Historically, this group of patients has been excluded from clinical trials, relying on the extrapolation of results for clinical application in those aged 75 years or older. We sought to determine if older adults diagnosed with incurable gastroesophageal malignancies tolerated chemotherapy as well as younger patients, achieved similar survival outcomes, and to determine if patterns in rationale for non-receipt of chemotherapy in the elderly existed. In our retrospective analysis, we observed that older adults tolerated palliative chemotherapy as well as patients aged <75 years and achieved similar survival outcomes, suggesting that age itself should not be a determinant for receipt of palliative chemotherapy, with performance status and comorbidities more appropriate predictors of outcome.

## Introduction

Carcinoma of the stomach and esophagus contributes to significant global cancer-related morbidity and mortality. Gastric cancer is the 5th most common malignancy and 3rd leading cause of cancer-related deaths, while esophageal cancer is the 8th most common malignancy and 6th leading cause of cancer-related death.^1-3^ The incidence of gastroesophageal junction carcinomas is on the rise, especially in those aged >65 years.^4^ Sixty percent of patients diagnosed with either gastric or esophageal carcinoma (GEC) are aged ≥65 years with the incidence expected to increase 67% by the year 2030.^5,6^ The median age at diagnosis for GEC based on the SEER database is approximately 68 in Europe and greater than 70 in the UK.^7,8^ Furthermore, 26% of patients diagnosed with cancer in the US are aged >75 years, yet only 10% of participants in U.S. National Cancer Institute Cooperative Group clinical trials are >75 years.^9^

Exclusion based on age is no longer endorsed, with 2 pooled analyses comparing outcomes in trials between young and old adults suggesting that age itself is not a determinant on outcome or toxicity.^10,11^ In spite of this, older patients are often undertreated. The seminal phase III clinical trials for GEC, whilst stating patients aged ≥75 are eligible, included very few elderly patients, with the median age 60-65.^12-17^ Real-world data have consistently shown that patients are generally older and only around 75% of patients actually receive palliative therapy highlighting the need to report real-world data in order to demonstrate clinical effectiveness as well as toxicity patterns.^18-21^ We sought to determine survival outcomes and the incidence of toxicity in older adults diagnosed with metastatic GEC receiving palliative chemotherapy, comparing those aged 64-74 to those aged ≥75 years.

## Materials and methods

### Study population and data collection

Patients with either recurrent or de novo metastatic GEC diagnosed between 2007-2019 at Princess Margaret Cancer Centre, Toronto, Canada were identified from an institutional registry. Non-curative, locally advanced patients were excluded. All patients were aged 65 years and older. The histologic subtypes adenocarcinoma and squamous cell carcinoma were included. Patient characteristics including age, gender, ethnicity, body mass index (BMI), Charlson co-morbidity index (CCI), and Eastern Cooperative Oncology Group (ECOG) performance status were recorded. The CCI score was calculated by summating a score associated with patient comorbidities (myocardial infarction (1), congestive heart failure (1), peripheral vascular disease (1), cerebrovascular accident or transient ischaemic attack (1), chronic obstructive pulmonary disease (1), connective tissue disorder (1), peptic ulcer disease (1), liver disease (mild (1)/moderate or severe (2)), diabetes mellitus (uncomplicated (1)/end-organ damage (2)), hemiplegia (2), moderate or severe chronic kidney disease (2), other solid tumour (2), leukaemia (2), lymphoma (2), or acquired immunodeficiency syndrome (6)).^22^

Patients were grouped into age brackets based on the National Institute of Ageing at the U.S. National Institute of Health classification of age: “young-old” (65-74 years) and “older-old” (≥ 75 years).^9,23^ Tumor characteristics including the site of primary tumor, histologic type, tumor grade, metastatic sites involved, and human epidermal growth factor receptor 2 (HER2) status were recorded, with HER2 positivity determined by either immune-histochemistry (IHC) 3 + or in-situ hybridization (ISH) amplification. Treatment type was collected including chemotherapy (yes/no), chemotherapy regimen, number of chemotherapy agents administered, number of lines of treatment, palliative radiation (yes/no), surgery (yes/no), or best supportive care (yes/no). The primary explanation for non-receipt of chemotherapy was determined as per the clinician notes recorded in institutional electronic records and was either age, ECOG performance status, CCI, or patient refusal.

Clinical staging was as per the American Joint Committee on Cancer (AJCC) 8th edition. In line with the AJCC 8th edition classification, carcinomas of the upper gastroesophageal junction (AEG1-2) were grouped with esophageal carcinoma, and carcinoma of the lower gastroesophageal junction (AEG3) were grouped with gastric carcinoma. The research was reviewed and approved by the Institutional Research Ethics Board (Princess Margaret Cancer Centre, University Health Network, Toronto, Canada; REB Approval Number: 20-5025) and was performed in accordance with the principles of the Declaration of Helsinki Good Clinical Practice.

### Statistical analysis

Descriptive statistics were used to compare differences in the distribution of covariates and age group. Time-to-event data were analyzed using the Kaplan-Meier curves and compared using log-rank tests. Progression-free survival (PFS) and overall survival (OS) were calculated from the date of diagnosis of metastatic disease to the date of first progression and date of death or date lost-to-follow-up, respectively. In addition, outcomes were compared using predefined multivariable Cox proportional hazards regression models, adjusting for gender, CCI (0 vs 1 vs 2 vs 3+), ethnicity (Non-Asian vs Asian), tumor histology (adenocarcinoma vs squamous cell carcinoma), primary location (esophagus/AEG1-2 vs gastric/AEG3), and ECOG performance status (0 vs 1 vs 2+). A subset multivariate analysis for patients with chemotherapy as first-line of treatment was also assessed. Statistical analyses were performed using R version 3.5.0 (R Core Team 2018, Vienna, Austria). All *P*-values were 2 sided and <.05 was considered statistically significant.

## Results

Three hundred and seven patients were identified in the analysis, 198 “young-old” and 109 “older-old.” Cohort demographics are shown in Table 1. At the time of diagnosis, the median age for “young-old” was 70.0 years and 79.5 years for “older-old” (*P* < .001). There was no statistically significant difference between age groups in respect to gender (*P* = .89), ethnicity (*P* = .21), BMI (*P* = .71) and ECOG *P*erformance status (*P* = .087). One hundred and three (52%) “young-old” and 31 (28%) “older-old” patients had a CCI of 0 (*P* < .001). 167 (84%) “young-old” and 91 (83%) “older-old” patients had a histological diagnosis of adenocarcinoma (*P* = .87), with the primary location of the tumor for “young-old” patients predominately esophageal/AEG1-2 (62%) compared to 48% in “older-old” patients (*P* = .022). One hundred forty-two (72%) “young-old” and 69 (63%) “older-old” patients had de novo metastatic disease at initial diagnosis, with the median number of metastatic sites three for both “young old” and “older-old,” ranging from 1 to 6 metastatic sites for both groups (*P* = .64). Peritoneal metastases were present at diagnosis in 22% “young-old” and 32% “older-old” patients (*P* = .39). There was no difference in the incidence of different tumor grades, with poorly differentiated carcinoma (grade 3) most common in both age groups (*P* = .92). Forty-five (23%) “young-old” patients and 16 (15%) ‘older-old’ patients were HER2 positive (*P* = .005).

**Table 1.** Baseline clinicopathologic characteristics.

Characteristic	Age 65-74 years	Age ≥75 years	*P*-value
	(*n* = 198)	(*n* = 109)	
Age (median) (years)	70.0 (65,74.9)	79.5 (75.2,92.4)	<.001
*Gender*			.89
Male	147 (74%)	82 (75%)	
Female	51 (26%)	27 (25%)	
*Ethnicity*			.21
Asian	22 (11%)	18 (17%)	
Non-Asian	176 (89%)	91 (83%)	
BMI (median)	24.4 (13,42.7)	24.5 (16.5,37.7)	.71
*CCI (groups)*			<.001
0	103 (52%)	31 (28%)	
1	37 (19%)	30 (28%)	
2	34 (17%)	29 (27%)	
3+	24 (12%)	19 (17%)	
*ECOG (groups)*			.16
0	37 (19%)	17 (18%)	
1	101 (52%)	46 (43%)	
2+	58 (30%)	43 (41%)	
*Histology*			.87
Adenocarcinoma	167 (84%)	91 (83%)	
Squamous cell carcinoma	31 (16%)	18 (17%)	
*Location*			.022
Esophagus/AEG1-2	122 (62%)	52 (48%)	
Gastric/AEG-3	76 (38%)	57 (52%)	
*Grade*			.92
G1	10 (5%)	5 (5%)	
G2	46 (23%)	28 (26%)	
G3	91 (46%)	46 (42%)	
GX (unknown)	51 (26%)	30 (28%)	
*HER2 status*			.005
Negative	87 (44%)	36 (33%)	
Positive	45 (23%)	16 (15%)	
Unknown	66 (33%)	57 (52%)	
*Previous surgery*			.83
No	151 (76%)	73 (67%)	
Yes	47 (24%)	36 (33%)	
*Metastases*			.16
Synchronous	142 (72%)	69 (63%)	
Recurrent	56 (28%)	40 (37%)	
Number metastatic sites	3 (1,6)	3 (1,6)	.64
(Median)			
*Number metastatic sites (n)*			.39
1	26 (13%)	15 (14%)	
2	37 (19%)	27 (25%)	
3+	135 (68%)	67 (61%)	

Abbreviations: BMI, body mass index; CCI, Charlson co-morbidity index; ECOG, European clinical oncology group performance status; AEG, Siewert classification esophagastric junction cancers; HER2, human epidermal growth factor receptor 2.

One hundred and nineteen (60%) “young-old” and 25 (23%) “older-old” patients received palliative chemotherapy (*P* < .001; Table 2). Thirty-seven (19%) “young-old” and 31 (28%) “older-old” patients received palliative radiation therapy whilst 34 (17%) and 49 (45%) patients respectively received best supportive care. The median number of chemotherapy agents used in first-line treatment was three (1,4) for “young-old” and two (1,3) for “older-old” patients (*P* = .002), with a total of 24 different chemotherapy regimens administered (Supplementary Table S1). 15 chemotherapy regimens (62%) contained a platinum chemotherapy, whilst 19 (79%) contained two or more anti-cancer drugs. Seven “young-old” and one ‘older-old’ patient received first-line palliative chemotherapy on a clinical trial. Seven patients received either targeted treatment for HER2 positive carcinoma (trastuzumab (*n* = 3); zanidatamab (*n* = 1)) or immune checkpoint inhibitors (ICI) (nivolumab (*n* = 2); pembrolizumab (*n* = 1)). All of these patients received targeted treatment of ICI in combination with chemotherapy. Performance status was the primary listed explanation for non-receipt of chemotherapy for both “young-old” (*n* = 36; 46%) and “older-old” (*n* = 30; 36%) patients. CCI was the documented reason in 11 (14%) and 8 (10%) of patients respectively. Patient refusal accounted for 24% (*n* = 19) and 21% (*n* = 18) of non-receipt of chemotherapy in “young-old” and ‘older-old’ respectively. Importantly, age was not considered a prohibitive cause in “young-old” patients (*n* = 0), whilst it was the documented explanation in 21 (25%) “older-old” patients (*P* < .001). A non-significant difference in patients receiving palliative radiation therapy was observed between “young-old” (*n* = 103; 52%) and “older-old” (*n* = 42; 39%) patients (*P* = .051).

**Table 2.** Palliative treatment received for “young-old” and ‘older-old’ patients.

Covariate	Age 65-74 years	Age ≥75 years	*P*-value
	(*n* = 198)	(*n* = 109)	
Palliative treatment			<.001
Chemotherapy	119 (60%)	25 (23%)	
Radiation only	37 (19%)	31 (28%)	
BSC	34 (17%)	49 (45%)	
Surgery	4 (2%)	3 (3%)	
Unknown	4 (2%)	1 (1%)	
Reason chemotherapy omitted			<.001
Age	0 (0%)	21 (25%)	
CCI	11 (14%)	8 (10%)	
ECOG	36 (46%)	30 (36%)	
Patient refused	19 (24%)	18 (21%)	
Unknown	13 (16%)	7 (8%)	
Number of chemotherapy agents (1st line) (median)	3 (1,4)	2 (1,3)	.002
Reason 1st line chemotherapy stopped			.97
Clinician choice	17 (14%)	3 (12%)	
Disease Progression	78 (66%)	16 (64%)	
Patient choice	3 (3%)	1 (4%)	
Toxicity	17 (14%)	3 (12%)	
Unknown	4 (3%)	2 (8%)	
2nd line chemotherapy			<.001
Yes	64 (32%)	6 (6%)	
No	129 (65%)	100 (92%)	
Unknown	5 (3%)	3 (2%)	
3rd line chemotherapy			.005
Yes	19 (10%)	2 (2%)	
No	165 (83%)	104 (95%)	
Unknown	14 (7%)	3 (3%)	
RT at any stage			.051
Yes	103 (52%)	42 (39%)	
No	92 (46%)	56 (51%)	
Unknown	3 (2%)	11 (10%)	

Abbreviations: BSC, best supportive care; BMI, body mass index; CCI, Charlson co-morbidity index; ECOG, European clinical oncology group performance status; RT, radiation therapy.

The primary explanation for cessation of 1st-line palliative chemotherapy was disease progression for both “young-old” (*n* = 78; 67%) and “older-old” (*n* = 16; 70%) with toxicity the reasoning in 17 (15%) “young-old” and 3 (13%) “older-old” patients (*P* = .97) (Table 2). Relative to “older-old” patients, ‘young-old’ was more likely to receive 2nd-line (32% vs 6%; *P* < .001) or 3rd-line (10% vs 2%; *P* = .005) chemotherapy (Table 2).

**Figure 1.**
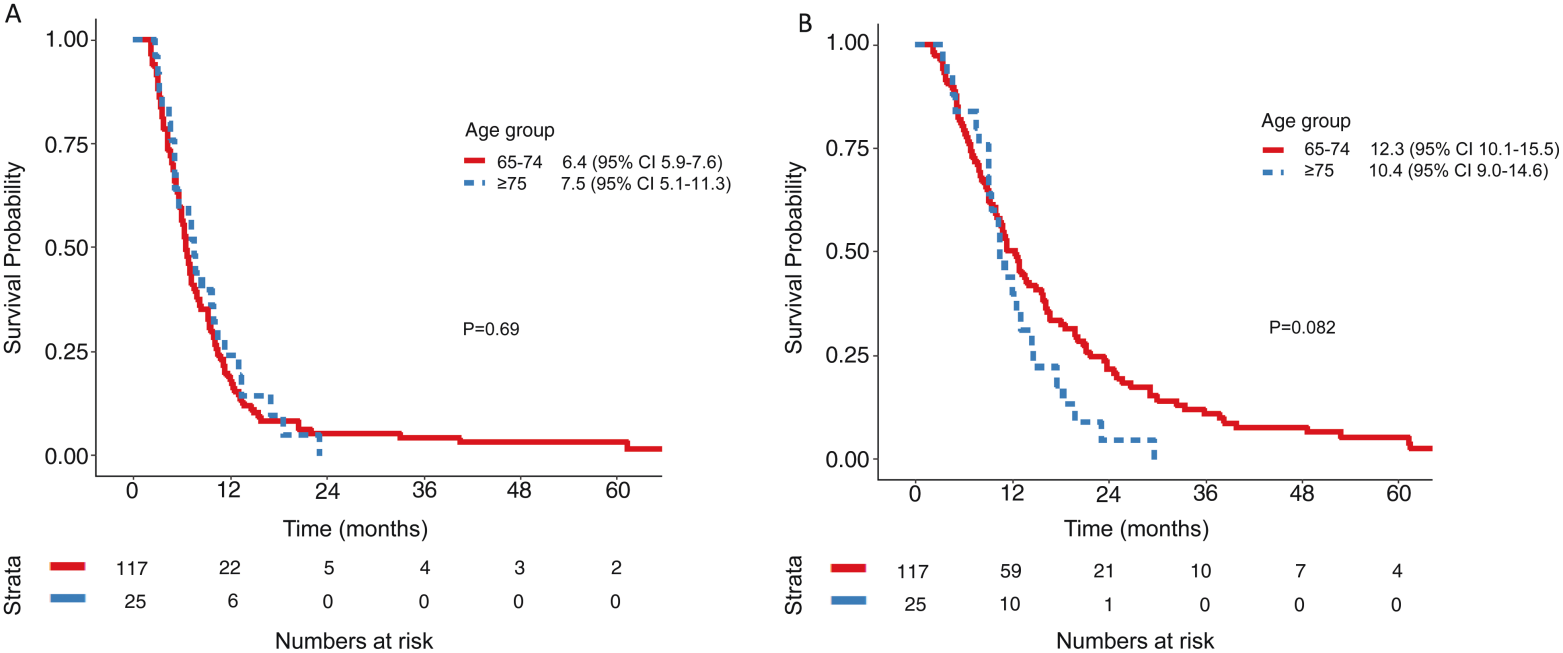
Kaplan-Meier curves for progression-free (PFS) (A) and overall survival (OS) (B) in patients receiving palliative chemotherapy (*n* = 144). (A) Median PFS “young-old” (65-74 years) 6.4m (95% CI 5.9-7.6), median PFS “older-old” (≥ 75 years) 7.5m (95% CI 5.1-11.3) (*P* = .69). (B) Median OS “young-old” 12.3m (95% CI 10.1-15.5), median OS “older-old” 10.4m (95% CI 9.0-14.6) (*P* = .082).

**Figure 2.**
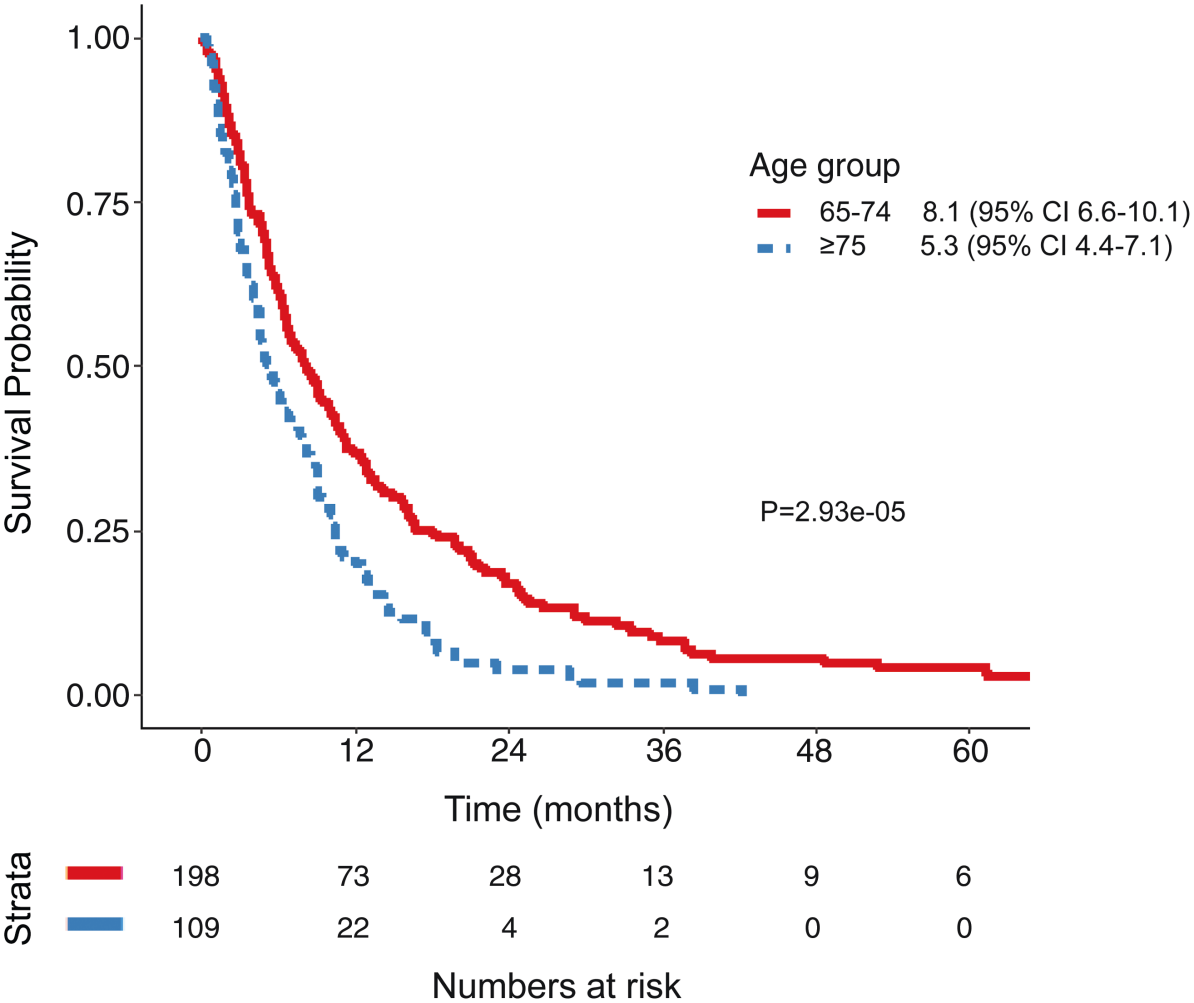
Overall survival (OS) Kaplan-Meier curve for total population (*n* = 307) median OS “young-old” (65-74 years) 8.1m (95% CI 6.6-10.1), median OS “older-old” (≥ 75 years) 5.3m (95% CI 4.4-7.7; *P* = 2.93e−05 log rank).

Observed PFS in “young-old” patients receiving palliative chemotherapy was 6.4 months (95% CI 5.9-7.6) and 7.5 months (95% CI 5.1-11.3) in ‘older-old’ patients (*P* = .69; Table 3; Figure 1A). The respective OS was 12.3 months (95% CI 10.1-15.5) and 10.4 months (95% CI 9.0-14.6) (*P* = .082) (Figure 1B). Among patients who received best supportive care (BSC) only, OS was similar, with 2.2 months (95% CI 1.6-3.2) in the “young-old” and 2.7 months (95% CI 2.2-4.4) in the ‘older-old’ patients. Observed OS in the entire population, including those that did not receive palliative chemotherapy, was longer in the “young-old” cohort (8.1m, 95% CI 6.6-10.1) compared to “older-old” (5.3m, 95% CI 4.4-7.7) (*P* = 2.93e−05 Log Rank; Figure 2).

**Table 3.** Survival outcomes.

	Age 65-74 years, months (95% CI)	Age ≥ 75 years, months (95% CI)	*P*-value
Progression-free survival with chemotherapy	6.4 (5.9-7.6)	7.5 (5.1-11.3)	.69
Overall survival with chemotherapy	12.3 (10.1-15.5)	10.4 (9.0-14.6)	.082
Overall survival with radiation only	4.8 (3.6-6.5)	6.4 (4.3-10.4)	.22
Overall survival with BSC	2.2 (1.6-3.2)	2.7 (2.2-4.4)	.19
Overall survival entire cohort	8.1 (6.6-10.1)	5.3 (4.4-7.1)	.000029

Abbreviations: BSC, best supportive care; CTx, chemotherapy.

Multivariate analysis between “young-old” and “older-old” cohorts receiving palliative chemotherapy identified that CCI was predictive for PFS with the HR for CCI one and 2 compared to zero 0.67 (95% CI 0.42-0.94) and 0.58 (95% CI 0.36-0.94) respectively, while CCI 3 + had an HR 1.40 (95% CI 0.66-2.97) (*P* = .039). No other pre-selected variables were predictive of PFS. ECOG performance status was predictive for OS, with the HR 1.85 (95% CI 1.03-3.33) for ECOG 2 + compared to ECOG 0 (*P* = .031; Table 4). All other variables were not predictive for OS. When analyzing the entire population (*n* = 307), both receipt of chemotherapy and ECOG performance status predicted PFS and OS (Supplementary Table S2).

**Table 4.** Multivariate cox proportional hazard analysis for progression-free survival and overall survival for patients treated with chemotherapy.

Covariate, *n* (events)	HR (95% CI)	*P*-value	Global *P*-value
Progression-free survival			
Age, 139 (134)		.94	.94
65-75	Reference		
>75	1.02 (0.64-1.63)		
Gender, 139 (134)		.35	.35
Female	Reference		
Male	1.22 (0.81-1.86)		
CCI, 139 (134)			.039
0	Reference		
1	0.67 (0.42-1.05)	.083	
2	0.58 (0.36-0.94)	.026	
3+	1.40 (0.66-2.97)	.38	
Ethnicity, 139 (134)		.58	.58
Non-Asian	Reference		
Asian	1.16 (0.69-1.94)		
Histology, 139 (134)		.78	.78
Adenocarcinoma	Reference		
Squamous cell carcinoma	0.92 (0.50-1.69)		
Location primary tumour, 139 (134)		.7	.7
Esophagus/AEG1-2			
Gastric/AEG3	Reference		
	1.08 (0.72-1.62)		
ECOG, 139 (134)			.26
0	Reference		
1	1.16 (0.75-1.80)	.51	
2+	1.59 (0.90-2.79)	.11	
Overall survival			
Age, 139 (128)		.057	.057
65-74	Reference		
≥75	1.62 (0.98-2.66)		
Gender, 139 (128)		.7	.7
Female	Reference		
Male	1.09 (0.71-1.68)		
CCI, 139 (128)			.35
0	Reference		
1	0.79 (0.50-1.27)	.34	
2	0.83 (0.51-1.35)	.45	
3+	1.65 (0.73-3.71)	.23	
Ethnicity, 139 (128)		.5	.5
Non-Asian	Reference		
Asian	0.83 (0.49-1.42)		
Histology, 139 (128)		.97	.97
Adenocarcinoma	Reference		
Squamous cell carcinoma	0.99 (0.49-1.86)		
Location primary tumour, 139 (128)		.87	.87
Esophagus/AEG1-2			
Gastric/AEG3	Reference		
	1.03 (0.68-1.56)		
ECOG, 139 (128)			.031
0	Reference		
1	0.94 (0.59-1.50)	.78	
2+	1.85 (1.03-3.33)	.04	

Abbreviations: AEG, Siewert classification esophagastric junction cancers; CCI, Charlson co-morbidity index; ECOG, European clinical oncology group performance status.

## Discussion

International guidelines recommend systemic therapy with a platinum–fluoropyrimidine doublet as the chemotherapy backbone in metastatic GEC.^24,25^ Our analysis suggests that this observed benefit occurs irrespective of age, and that age should not independently determine receipt of chemotherapy. This observation is similar to previous publications analyzing outcomes in lung cancer, early stage breast cancer and locally advanced esophageal cancer treated with chemoradiation.^26-28^ A pooled analysis of 8 consecutive North Central Cancer Group (NCCTG) trials in GEC indicated that patients ≥ 65 who received palliative chemotherapy had comparable survival outcomes to that observed in younger adults, albeit with increased toxicity.^11^ In our cohort, among patients who received palliative systemic therapy, median OS was 12.3 and 10.4 months in the “young-old” and “older-old” groups, respectively. These survival outcomes are comparable to first-line systemic therapy phase III trials.^17,29,30^ Despite similar outcomes, there is a notable difference between “young-old” and ‘older-old’ patients with respect to receipt of subsequent lines of systemic therapy beyond the first. While 32% of “young-old” patients who received first-line therapy received subsequent lines of systemic therapy, only 6% of ‘older-old’ patients received 2nd line treatment. This difference may have contributed to the observed separation of the OS survival curves (Figure 1B).

In our retrospective cohort, 77% of patients aged ≥75 did not receive systemic therapy with age being the primary reason in 25% of them. Survival was markedly different between those who received palliative therapy, with mOS of 10.4 months, compared to 2.7 months for those who received BSC only. This highlights the important role of the oncologist to make both evidence-based and patient-centered recommendations. Recent data from the GO2 study among patients who are deemed frail, with a median age of 76, and deemed unable to tolerate full-combination chemotherapy, a reduced-intensity regimen produced less toxic effects while having similar survival outcomes.^31^ Given the concern for increased toxicity in the elderly, oxaliplatin-based regimen appear to be both efficacious and well tolerated.^32-34^ Significant toxicity leading to treatment cessation was not observed to be different between “young-old” and “older-old” adults in our cohort. In light of our data suggesting that age should not be the sole determinant for withholding systemic therapy, a dose-reduced regimen would be preferable. Furthermore, although the GO2 study did not investigate the impact of co-morbidities, prior meta-analysis of solid organ malignancies have shown that a CCI score of 2 or less was associated with a more favorable outcome.^35^ As such, we propose that comorbidity and performance status are more predictive of survival and that age alone should not be a barrier for systemic therapy.

Recent clinical practice guidelines recommend geriatric assessment guided management should be considered in all patients over 65 years old.^36^ This assessment evaluates multiple domains including functional status, comorbidity, medications, cognition, fatigue, psychological status, nutrition, and frailty assessment. Development of streamlined care pathways and framework for how oncologists can incorporate geriatric assessment into clinical practice can avoid both over- and under-treatment. Furthermore, understanding which domains may contribute to treatment tolerability can help to provide more patient-centered recommendations for chemotherapy in the older patients. Two recent randomized clinical trials demonstrated that the integration of geriatric assessment can reduce 10-20% of serious chemotherapy-related toxic effects.^37,38^ Recent expert panel recommends the Practical Geriatric Assessment instrument (https://society.asco.org/sites/new-www.asco.org/files/content-files/practice-patients/documents/2023-PGA-Final.pdf) as the standard for geriatric assessment.^36^

## Limitations

There are important limitations to our real-world study. It is a retrospective review of a single high-volume center experience. Even at a quaternary referral center, the total number of “older-old” patients remains small and conclusions made regarding survival need to be interpreted with caution. This underscores the importance of a concerted effort to collect real-world data on the older patients. In our cohort, there is likely a selection bias of more “fit” or “motivated” patients being referred to a quaternary referral center compared to the “average” patient being treated in the community. Furthermore, although a detailed review was conducted, reason for omitting therapy was likely multifactorial. Practical geriatric assessments were not collected for the older patients which will need to be studied in the future to better understand which domains are impacted and how to better select patients for therapy.

In light of the GO2 study, chemotherapy dosing was not collected, and this will need to be reported in future real-world studies to determine the association between dosing and outcomes. Given the long duration of this study, chemotherapy backbone has also evolved from the more toxic triplet to a platinum–fluoropyrimidine doublet.^39^

Despite these shortcomings, this study represents a large, well-curated dataset of metastatic GEC with detailed clinical information including CCI and treatments received. Given the pace of advances in the field, the standard of care has evolved over the past decade. There is growing evidence that GEC is enriched with several actionable targets including HER2, mismatch repair deficiency, PD-L1 expression, as well as emerging targets such as Claudin 18.2 and FGFR2b.^17,40-43^ Many of these pivotal trials had very few “older-old” patients; however, these results are likely to be extrapolated in clinical practice. Future studies will need to take into consideration of different toxicity profiles of targeted agents and chemo-immunotherapy when investigating real-world outcomes as it relates to older patients.

## Conclusion

Our real-world study demonstrates that a significant number of “older-old” GEC patients do not receive systemic chemotherapy. Among the subset of “older-old” patients who do receive treatment, survival was comparable with ‘young-old’ patients, suggesting that age itself should not be the sole barrier to receipt of palliative systemic therapy. While concurrent comorbidities were associated with a reduced median PFS, age did not influence survival outcomes, nor was there a demonstratable difference in significant toxicity leading to treatment cessation. Geriatric assessment-guided management of older patients with GEC is recommended to ensure appropriate patient selection for palliative systemic therapy to maximize survival while minimizing toxicity.

## Supplementary material

Supplementary material is available at *The Oncologist* online.

oyae190_suppl_Supplementary_Tables

## Data Availability

The data underlying this article will be shared on reasonable request to the corresponding author.
