# Supplementary material for: Outcomes in older adults with metastatic esophageal and gastric carcinoma treated with palliative chemotherapy
Source: Oncologist. 2024 Jul 24;29(11):e1501–10. doi: 10.1093/oncolo/oyae190 (PMC11546644; doi:10.1093/oncolo/oyae190)
Supplement: oyae190_suppl_Supplementary_Tables [file oyae190_suppl_supplementary_tables.docx]

**Supplementary Table S1: Systemic treatment regimens administered to patient population**

|  | **65-74 years**  **(n=116)^*^** | **≥75 years**  **(n=25)** |
| --- | --- | --- |
| Capecitabine | 0 (0) | 5 (20) |
| Capecitabine + Irinotecan + Oxaliplatin | 1 (1) | 0 (0) |
| Capecitabine + Oxaliplatin (CAPOX) | 1 (1) | 0 (0) |
| Capecitabine + Trastuzumab | 1 (1) | 1 (4) |
| Carboplatin + Paclitaxel | 4 (3) | 0 (0) |
| Cisplatin + Capecitabine | 11 (9) | 3 (12) |
| Cisplatin + Capecitabine + Trastuzumab | 15 (13) | 2 (8) |
| Cisplatin + Capecitabine +/- Ramucirumab (clinical trial) | 1 (1) | 0 (0) |
| Cisplatin + Fluorouracil | 7 (6) | 1 (4) |
| Cisplatin + Fluorouracil + Trastuzumab | 8 (7) | 0 (0) |
| Cisplatin + Fluorouracil +/- Pembrolizumab (KEYNOTE-590 clinical trial) | 3 (3) | 0 (0) |
| Docetaxel | 0 (0) | 1 (4) |
| Epirubicin + Cisplatin + Capecitabine (ECX) | 19 (16) | 2 (8) |
| Epirubicin + Cisplatin + Fluorouracil (ECF) | 15 (13) | 2 (8) |
| Fluorouracil | 1 (1) | 0 (0) |
| Fluorouracil + Etoposide | 1 (1) | 0 (0) |
| Fluorouracil + Irinotecan (FOLFIRI) | 4 (3) | 1 (4) |
| Fluorouracil + Oxaliplatin + Docetaxel (FLOT) | 1 (1) | 0 (0) |
| Fluorouracil + Oxaliplatin (FOLFOX) | 18 (16) | 5 (20) |
| Fluorouracil + Oxaliplatin (FOLFOX) + Nivolumab + Relatlimab (CHECKMATE-060 clinical trial) | 1 (1) | 0 (0) |
| Fluorouracil + Oxaliplatin (FOLFOX) + Zanidatamab (ZWI-ZW25-201 clinical trial) | 0 (0) | 1 (4) |
| Ipilimumab + Nivolumab (CHECKMATE-649 clinical trial) | 1 (1) | 0 (0) |
| Paclitaxel | 2 (2) | 1 (4) |
| Tasquinimod (clinical trial) | 1 (1) | 0 (0) |

* Three (n = 3) patients did not have their first-line chemotherapy regimen recorded

**Supplementary Table S2: Multivariate cox proportional hazard analysis for progression-free survival and overall survival for the entire population**

| **Covariate, n (events)** | **HR (95% CI)** | **p-value** | **Global p-value** |
| --- | --- | --- | --- |
| **Progression-free Survival** | | | |
| **Age, 302 (294)**  65-75  ≥75 | Reference  1.00 (0.76-1.31) | 0.99 | 0.99 |
| **Gender, 302 (294)**  Female  Male | Reference  1.15 (0.87-1.53) | 0.33 | 0.33 |
| **CCI, 302 (294)**  0  1  2  3+ | Reference  0.97 (0.71-1.32)  0.89 (0.65-1.22)  0.72 (0.49-1.07) | 0.82  0.46  0.11 | 0.43 |
| **Ethnicity, 302 (294)**  Non-Asian  Asian | Reference  1.12 (0.78-1.60) | 0.54 | 0.54 |
| **Histology, 302 (294)**  Adenocarcinoma  Squamous cell carcinoma | Reference  0.75 (0.52-1.09) | 0.14 | 0.14 |
| **Location Primary tumour, 302 (294)**  Esophagus/AEG1-2  Gastric/AEG3 | Reference  1.04 (0.79-1.38) | 0.76 | 0.76 |
| **ECOG, 302 (294)**  0  1  2+ | Reference  1.45 (1.05-2.01)  2.67 (1.87-3.80) | 0.026  <0.001 | <0.001 |
| **Patients received chemotherapy as initial treatment, 302 (294)**  Yes  No | Reference  1.55 (1.18-2.02) | 0.0015 | <0.001 |
| **Overall survival** | | | |
| **Age, 302 (288)**  65-74  ≥75 | Reference  1.15 (0.87-1.53) | 0.32 | 0.32 |
| **Gender, 302 (288)**  Female  Male | Reference  1.12 (0.84-1.50) | 0.45 | 0.45 |
| **CCI, 302 (288)**  0  1  2  3+ | Reference  1.09 (0.79-1.50)  1.07 (0.77-1.49)  0.75 (0.50-1.13) | 0.59  0.67  0.17 | 0.33 |
| **Ethnicity, 302 (288)**  Non-Asian  Asian | Reference  1.00 (0.69-1.44) | 1.0 | 1.0 |
| **Histology, 302 (288)**  Adenocarcinoma  Squamous cell carcinoma | Reference  0.80 (0.55-1.17) | 0.25 | 0.25 |
| **Location Primary tumour, 302 (288)**  Esophagus/AEG1-2  Gastric/AEG3 | Reference  1.03 (0.78-1.36) | 0.85 | 0.85 |
| **ECOG, 302 (288)**  0  1  2+ | Reference  1.45 (1.04-2.04)  3.04 (2.11-4.39) | <0.001 | <0.001 |
| **Patients received chemotherapy as initial treatment, 302 (288)**  Yes  No | Reference  2.5 (1.88-3.32) | <0.001 | <0.001 |

***Abbreviations:*** CCI = Charlson co-morbidity index; ECOG = European clinical oncology group performance status; AEG = Siewert classification esophagastric junction cancers.
